# Reconstruction and analysis of a genome-scale metabolic model for *Scheffersomyces stipitis*

**DOI:** 10.1186/1475-2859-11-27

**Published:** 2012-02-23

**Authors:** Balaji Balagurunathan, Sudhakar Jonnalagadda, Lily Tan, Rajagopalan Srinivasan

**Affiliations:** 1 Institute of Chemical and Engineering Sciences, Agency for Science, Technology and Research, 1, Pesek Road, Jurong Island, Singapore 627833, Singapore; 2 Department of Chemical and Biomolecular Engineering, National University of Singapore, 10, Kent Ridge Crescent, Singapore 119260, Singapore

**Keywords:** Genome scale metabolic models, *Scheffersomyces stipitis*, Metabolic flux analysis, Xylose utilization, Anaerobic growth

## Abstract

**Background:**

Fermentation of xylose, the major component in hemicellulose, is essential for economic conversion of lignocellulosic biomass to fuels and chemicals. The yeast *Scheffersomyces stipitis *(formerly known as *Pichia stipitis*) has the highest known native capacity for xylose fermentation and possesses several genes for lignocellulose bioconversion in its genome. Understanding the metabolism of this yeast at a global scale, by reconstructing the genome scale metabolic model, is essential for manipulating its metabolic capabilities and for successful transfer of its capabilities to other industrial microbes.

**Results:**

We present a genome-scale metabolic model for *Scheffersomyces stipitis*, a native xylose utilizing yeast. The model was reconstructed based on genome sequence annotation, detailed experimental investigation and known yeast physiology. Macromolecular composition of *Scheffersomyces stipitis *biomass was estimated experimentally and its ability to grow on different carbon, nitrogen, sulphur and phosphorus sources was determined by phenotype microarrays. The compartmentalized model, developed based on an iterative procedure, accounted for 814 genes, 1371 reactions, and 971 metabolites. In silico computed growth rates were compared with high-throughput phenotyping data and the model could predict the qualitative outcomes in 74% of substrates investigated. Model simulations were used to identify the biosynthetic requirements for anaerobic growth of *Scheffersomyces stipitis *on glucose and the results were validated with published literature. The bottlenecks in *Scheffersomyces stipitis *metabolic network for xylose uptake and nucleotide cofactor recycling were identified by in silico flux variability analysis. The scope of the model in enhancing the mechanistic understanding of microbial metabolism is demonstrated by identifying a mechanism for mitochondrial respiration and oxidative phosphorylation.

**Conclusion:**

The genome-scale metabolic model developed for *Scheffersomyces stipitis *successfully predicted substrate utilization and anaerobic growth requirements. Useful insights were drawn on xylose metabolism, cofactor recycling and mechanism of mitochondrial respiration from model simulations. These insights can be applied for efficient xylose utilization and cofactor recycling in other industrial microorganisms. The developed model forms a basis for rational analysis and design of *Scheffersomyces stipitis *metabolic network for the production of fuels and chemicals from lignocellulosic biomass.

## Background

*Scheffersomyces stipitis *(*S. stipitis*), formerly known as *Pichia stipitis *[1], is a hemiascomycetous yeast, closely related to several yeast endosymbionts of passalid beetles that inhabit and decay white-rotted hardwood [2,3]. It has the highest native capacity for xylose fermentation of any known microbe [4,5]. Fed batch cultures of *S. stipitis *produce around 47 g/l of ethanol with yields of 0.36 g/g xylose at 30°C [4]. In addition to xylose, *S*. *stipitis *has the capability to ferment sugars from hydrolysates with yields equivalent to 80% of theoretical yield [6-8]. Auxotrophic strains have been created and methods for high efficiency transformation have been developed for *S. stipitis *[9,10]. Genetic tools based on a *loxP*/Cre recombination system have been developed for functional genomics and metabolic engineering of this yeast [11]. The availability of genetic tools and capability for fermentation of hydrolysates has made *S. stipitis *an attractive microorganism for bioconversion of lignocellulose to fuels and chemicals. *S. stipitis *has already been successfully engineered to produce lactic acid and xylitol [12,13]. However, *S. stipitis *suffers from some drawbacks like lower fermentation rates, lower tolerance to ethanol and absence of anaerobic growth [5,14,15].

As a parallel approach, xylose utilization pathway from *S. stipitis *has been used to engineer xylose metabolism in *Saccharomyces cerevisiae*. Successive cycles of metabolic engineering have improved xylose utilization in recombinant *S. cerevisiae *[16,17]. However, the ethanol productivity from xylose is still low. This has been attributed to: low substrate affinity of recombinant enzymes [18]; cofactor imbalance in the XR-XDH reactions [19,20]; low xylose transport capacity [21,22]; and failure to recognize xylose as a fermentable carbon source [23,24]. The holistic analysis of metabolism in *S. stipitis *could provide useful insights to identify shortcomings in *S. stipitis *and *S. cerevisiae *metabolic networks.

The complete genome of *S. stipitis *has been sequenced [25]. The functional annotation of the genome sequence showed numerous genes for lignocellulose bioconversion and systematic analysis of the genome sequence annotation is necessary to obtain useful insights. Genome scale metabolic models, which represent the link between the genotype and phenotype of the organism, can be reconstructed based on the genome sequence annotation and relevant biochemical and physiological information. These models have the ability to provide a holistic view of the metabolism of an organism. Once experimentally validated, these models can be used to characterize the metabolic resource allocation, generate experimentally testable predictions of cellular phenotypes, elucidate metabolic network evolution scenarios, design experiments that most effectively reveal the genotype-phenotype relationships, and design engineered microorganisms with desired properties like overproduction of commercially valuable chemicals [26-30]. Due to the genome wide-scale, these models enable systematic assessment of how perturbations in the metabolic network affect the organism as a whole which may not be possible by analyzing a set of enzymes or isolated pathways.

We have reported a framework for reconstruction of genome scale metabolic model of *S*. *stipitis *[31]. In this study, a genome scale metabolic model has been developed for *S. stipitis *based on the proposed framework and a recently published protocol [32]. Experimental procedure for the estimation of macromolecular composition of *S. stipitis *was standardized and used to obtain the biomass composition. Growth and non-growth associated maintenance energy requirements were also estimated from experimental data. The model was refined and validated based on the ability of *S. stipitis *to grow on different carbon, nitrogen, sulphur and phosphorus sources. In silico analysis of the model was used to identify biosynthetic requirements for anaerobic growth of *S. stipitis *in glucose and to analyze xylose utilization capability in *S. stipitis*. Model simulations were carried out to obtain insights on the recycling of nucleotide cofactors and mechanisms involved in mitochondrial respiration and oxidative phosphorylation.

## Results

### Reconstruction of the genome scale metabolic model for *Scheffersomyces stipitis*

**Figure 1:**
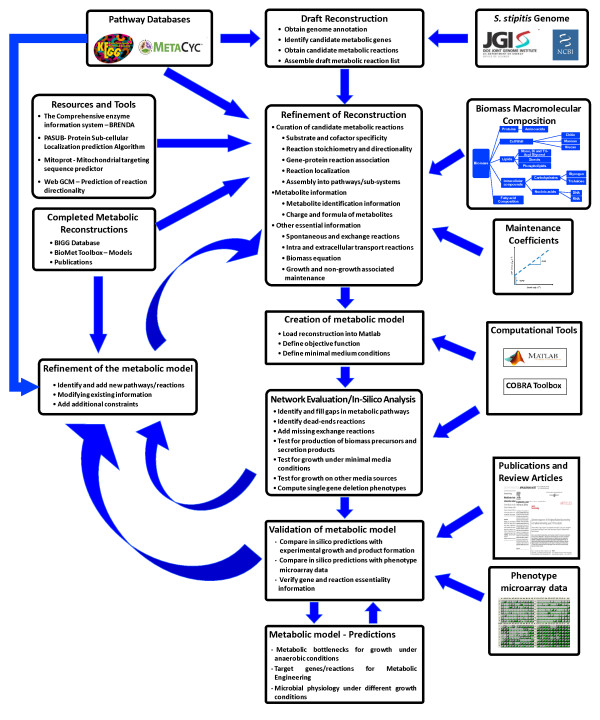
**Iterative procedure for reconstruction of the genome scale metabolic network of *Scheffersomyces stipitis***.

An initial metabolic reconstruction for *S. stipitis *was developed based on the protocol outlined in the methods section (Figure 1). Biomass macromolecular composition was experimentally determined. The constituents of biomass and the fractional contribution of these constituents to overall cellular biomass are summarized in Table 1. The growth and non-growth associated maintenance coefficient (GAM and NGAM) was estimated from growth rate and substrate uptake rate data. The details of the estimation are provided in the supplementary information (Additional File 1). The initial metabolic network consisted of 1167 reactions and this network was expanded to 1371 reactions based on high-throughput Biolog phenotyping data and metabolic gap analysis.

**Table 1:** Macromolecular composition of *S. stipitis *Biomass

Biomass Composition of *Scheffersomyces stipitis*			
**Metabolite**	**mmol/gDCW**	**Metabolite**	**mmol/gDCW**

**Amino Acids**		**DNA**	

Aspartic Acid	0.1566	dAMP	0.0112

Threonine	0.1809	dTMP	0.0112

Serine	0.2330	dGMP	0.0084

Glutamic Acid	0.3190	dCMP	0.0084

Glycine	0.4724	**RNA**	

Alanine	0.4735	AMP	0.0444

Cystine	0.0511	UMP	0.0522

Valine	0.2201	GMP	0.0361

Methionine	0.0559	CMP	0.0388

Iso-Leucine	0.1454	**Lipids**	

Leucine	0.2451	Sterol (ergosterol)	0.0560

Tyrosine	0.0741	**Phospholipids**	

Phenylalanine	0.1127	PhosphatidylInositol	0.0015

Histidine	0.1005	Phosphatidylethanolamine	0.0041

Lysine	0.2624	Phosphatidylcholine	0.0255

Arginine	0.1821	**Carbohydrates**	

Tryptophan	0.0248	Glycogen	0.2714

Proline	0.1592	Trehalose	0.0760

Asparagine	0.1511	Glucan	0.6107

Glutamine	0.1817	Mannan	0.7156

		Chitin	0.4528

### Characteristics of the reconstructed network

**Figure 2:**
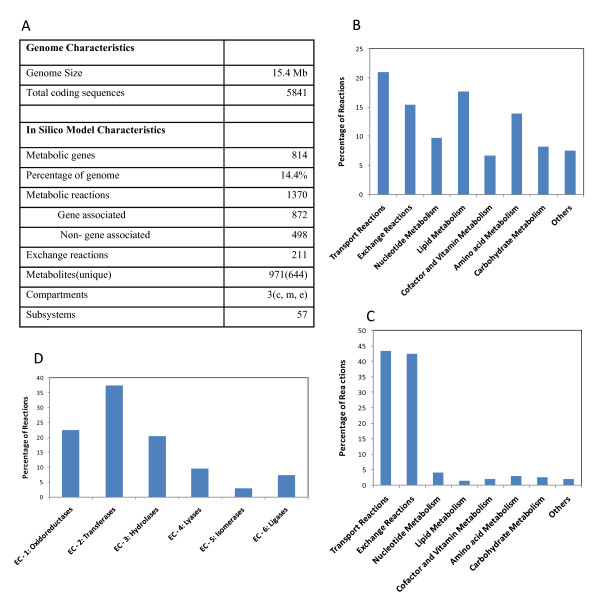
**Characteristics of the genome scale metabolic network**. **A**) Statistics. **B**) Functional classification of metabolic reactions in the model. **C**) Functional classification of the non-gene associated metabolic reactions in the model. **D**) Functional classification of enzyme classes in the model.

The characteristics of the reconstructed *S. stipitis *metabolic network are detailed in Figure 2. The complete reconstruction accounted for 814 open reading frames (ORFs) and consisted of 1370 reactions and 644 unique metabolites (Figure 2A). The details of the list of genes, reactions, metabolites and the GPR associations in the reconstruction are available as supplementary information (Additional File 2). The functional classification of the ORFs included in the reconstruction is summarized in Figure 2B. The reactions in the model were assigned to 57 different subsystems, organized into 8 groups. The number of non-gene associated reactions in each of these groups is shown in Figure 2C. The distribution of enzyme classes in the model is shown in Figure 2D.

**Figure 3:**
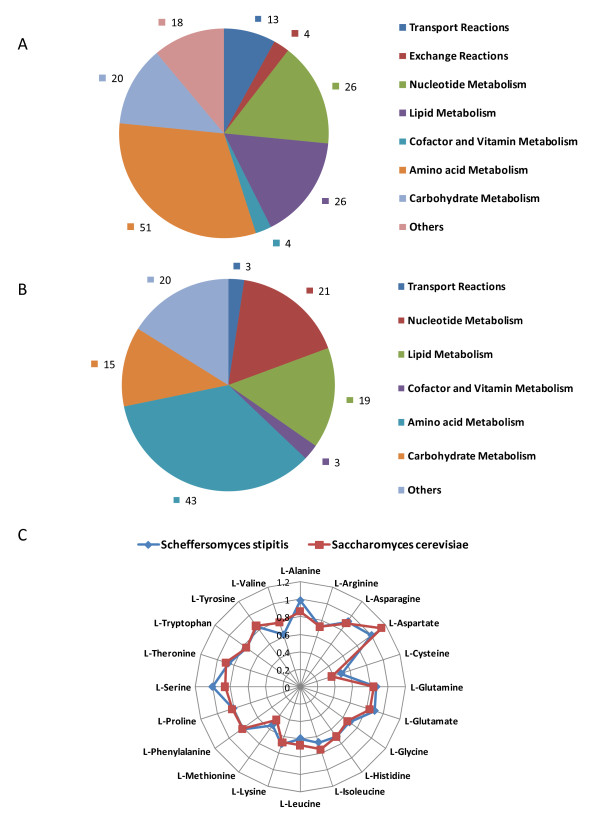
**Reaction essentiality, gene essentiality and amino acid production capability**. **A**) Functional distribution of the essential reactions in the model. **B**) Functional distribution of the essential genes in the model. **C**) Comparison of the amino acid production capability of *S. stipitis *and *S. cerevisiae *metabolic network on a carbon mole basis.

The basic capabilities of the in silico model to predict quantitatively the aerobic growth on glucose was determined. A growth demand function was formulated based on the estimated biomass composition detailing the required metabolites in the appropriate ratios. This demand function was used as the objective function for flux balance analysis. The number of genes and reactions essential for the production of biomass from a glucose based minimal media was computed. The distribution of essential reactions and essential genes among pathway groups is shown in Figure 3A and Figure 3B. The highest number of essential genes and reaction where associated with amino acid metabolism followed by nucleotide and lipid metabolism. The list of essential reactions is provided in supplementary information (Additional File 3). Capability to produce various amino acids from glucose was analyzed using the genome scale metabolic model and compared with that obtained with *S. cerevisiae *[33]. The theoretical yield of various amino acids on carbon mole basis is shown in Figure 3C. The yields obtained were comparable between the two yeasts, indicating the similarity in the biosynthetic networks for amino acid synthesis.

### Analysis of high-throughput substrate utilization

**Figure 4:**
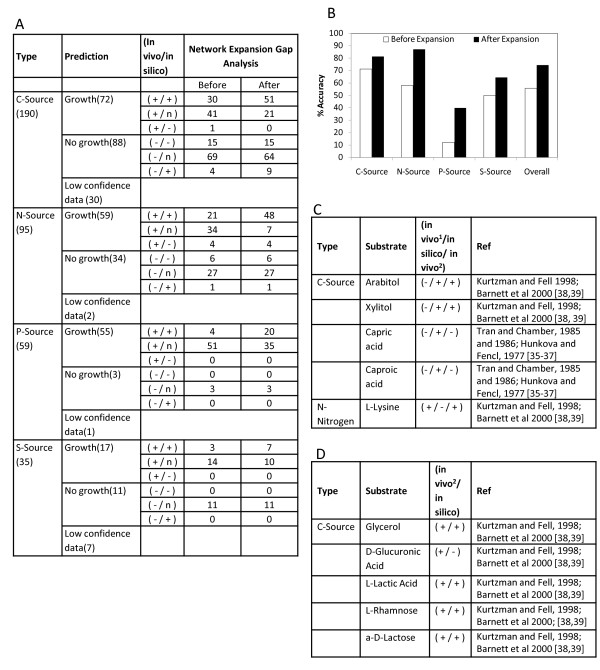
**Network expansion and metabolic gap analysis based on high-throughput substrate utilization data**. **A**) Comparison of experimental data from Biolog phenotype micro-arrays to model predictions across different substrate categories. Results are scored as + or - meaning growth or no growth determined from *in vivo*/*in silico *data. The n represents that corresponding pathway could not be included in the *S. stipitis *network due to unknown pathway enzymes. **B**) Improvement of prediction accuracy **C**) Comparison of incorrect predictions (+/- and -/+ cases in (**A**)) with published experimental results. (**D**) Comparison of *in silico *predictions with published experimental results for the Biolog substrates identified as low-confidence data. The Biolog data was considered as low confidence growth when the inference of growth/no-growth was difficult from the absorbance measurements. In vivo^1 ^from Biolog phenotyping, in vivo^2 ^from literature.

The in silico computations were compared with the high throughput phenotyping data from Biolog's Phenotype microarray technology [34] (Figure 4). 339 out of the 379 substrates tested (190 for carbon, 95 for Nitrogen, 59 for Phosphorus and 35 for Sulphur sources) were identified as data with sufficient confidence (Confidence of the data was estimated as described in the methods section) and were analyzed for consistency using the *S. stipitis *model. The list of substrates, confidence levels of data and model refinements are described in supplementary information (Additional File 4). Growth on substrates was simulated by fixing its specific uptake rate at 5 mmol/gDCW/h under aerobic conditions based on minimal media (Methods Section). The initial metabolic network reconstruction could predict qualitatively the outcome of Biolog data with 56% accuracy (189 in 339), but after network expansion and metabolic gap analysis, overall prediction efficiency was considerably improved to 74% (252 in 339) (Figure 4A). However, 14 disagreements (9 for carbon and 5 for nitrogen) were observed (Figure 4B); of these 5 cases were compared with experimental growth data available in literature for *S. stipitis *or related yeasts for corresponding substrates (Figure 4C).

Biolog phenotyping results indicated that capric acid and caproic acid cannot by utilized by *S*. *stipitis *as a sole carbon source, but the model predicted growth. However, it has been observed that capric, caproic and other fatty acids were known to inhibit the growth of *S*. *stipitis *[35,36] and other yeasts [37]. Since the inhibition mechanisms are not incorporated in the metabolic model, the in silico computations predicted growth on these substrates. In the case of nitrogen source utilization, Biolog phenotyping results and experimental data reported by [38,39] indicated growth on lysine as a sole nitrogen source. However, the in silico predictions did not predict growth as the pathway enzymes involved in metabolism of lysine has not been identified in *S. stipitis *genome. The in silico growth predictions for Biolog substrates evaluated as low confidence data were also compared with data available in literature for corresponding substrates. Experimental data was available from literature [38,39] for 5 out of 40 low-confidence cases and the model could correctly predict the utilization of these substrates with 80% accuracy (4 cases)(Figure 4D). Incorrect prediction for glucuronic acid was due to the lack of homologs for pathway enzymes. The examples of lysine and glucuronic acid utilization illustrate the capability of the model to pinpoint potential gaps in the understanding of metabolism and to guide experimental design.

### Metabolic requirements for anaerobic growth of *Scheffersomyces stipitis*

One drawback for using *S. stipitis *in industrial fermentation is its inability to grow under anaerobic conditions. The model developed was used to analyze the requirements for anaerobic growth. The model was simulated for anaerobic growth on a glucose based minimal media by reducing the oxygen uptake rate to zero, with an unconstrained uptake of sterols and unsaturated fatty acids (which have a known biosynthetic requirement for oxygen). No growth was predicted by the model simulations under these conditions. Growth under anaerobic conditions is a complex process. Several requirements need to be met for growth including biosynthetic requirements for oxygen, energy requirements, redox balance requirements and regulatory requirements.

While performing the model simulations, even though sterol and unsaturated fatty acids were added there could be other metabolites or biomass constituents in yeasts that requires oxygen for their biosynthesis. To identify the biosynthetic requirements for anaerobic growth, reaction insertion analysis was performed on the model using the reference metabolic database KEGG [40]. An initial set of metabolic reactions (802 reactions) were compiled from the KEGG database that is made up of only metabolites present in the model. These reactions were then inserted one at a time to determine their effect on growth under anaerobic conditions. Reactions already present in the model were ignored. Single reaction additions that resulted in a positive biomass flux were identified. The list of reactions leading to a positive biomass flux is summarized in Table 2. There were 28 such reactions, 12 of these reactions directly resulted in oxygen production. 10 reactions resulted in oxygen production through the formation of H_2_O_2 _(either directly or through glutathione or pyridoxine) and the remaining 6 reactions were selected for further analysis. Four of these reactions involve phospholipids and the stoichiometry of these reactions in the KEGG database is different from the way lipid metabolism reactions are represented in the metabolic model. The remaining two reactions convert dihydroorotate to orotate. The URA1 gene from *S*. *cerevisiae*, which converts dihydroortate to orotate with fumarate added to the medium as electron acceptor, was reported to result in enhanced anaerobic growth in *S. stipitis *[15]. This serves as a validation of the model developed for *S. stipitis*.

**Table 2:** List of reactions that lead to anaerobic growth on glucose identified by single reaction insertion analysis

S.NO	R Numbers	Reaction Formula	Biomass Flux	Reaction Flux
1	R00090*	h2o2[c] + h[c] + nadh[c] < = > 2 h2o[c] + nad[c]	0.4049	-20.0000

2	R00094*	nad[c] + 2 gthrd[c] < = > h[c] + nadh[c] + gthox[c]	0.4049	20.0000

3	R00113*	h2o2[c] + h[c] + nadph[c] < = > 2 h2o[c] + nadp[c]	0.4491	-20.0000

4	R00115*	nadp[c] + 2 gthrd[c] < = > h[c] + nadph[c] + gthox[c]	0.4491	20.0000

5	R00211^$ ^	o2[c] + pyr[c] + coa[c] < = > h2o2[c] + accoa[c] + co2[c]	0.2279	-2.6049

6	R00319^$ ^	o2[c] + lac-L[c] < = > h2o[c] + ac[c] + co2[c]	0.6300	-20.0000

7	R00360^$ ^	o2[c] + mal-L[c] < = > oaa[c] + h2o2[c]	0.2079	-0.1820

8	R00475^$ ^	o2[c] + glyclt[c] < = > glx[c] + h2o2[c]	0.2038	-0.0225

9	R00481^$ ^	asp-L[c] + o2[c] < = > h2o2[c] + iasp[c]	0.2055	-0.1579

10	R00500*	2 gthrd[c] < = > gthox[c]	0.6061	20.0000

11	R00533^$ ^	h2o[c] + o2[c] + so3[c] < = > h2o2[c] + so4[c]	0.2133	-0.2130

12	R00846^$ ^	o2[c] + glyc3p[c] < = > h2o2[c] + dhap[c]	0.2075	-0.1817

13	R01712*	pyr[c] + pydam[c] < = > ala-L[c] + pydx[c]	0.4073	20.0000

14	R01713*	oaa[c] + pydam[c] < = > asp-L[c] + pydx[c]	0.4049	20.0000

15	R01769^$ ^	h2o[c] + o2[c] + hxan[c] < = > h2o2[c] + xan[c]	0.2079	-0.1820

16	R01797^# ^	h2o[c] + cdpdag[c] < = > pa[c] + cmp[c]	17.8571	-7.4901

17	R01799^# ^	pa[c] + ctp[c] < = > ppi[c] + cdpdag[c]	17.8571	7.5572

18	R01800^# ^	ser-L[c] + cdpdag[c] < = > cmp[c] + ps[c]	0.2073	-0.0005

19	R01866^# ^	nadp[c] + dhor-S[c] < = > h[c] + nadph[c] + orot[c]	0.2062	0.0228

20	R01869^# ^	nad[c] + dhor-S[c] < = > h[c] + nadh[c] + orot[c]	0.2056	0.0227

21	R01879^$ ^	akg[c] + o2[c] + duri[c] < = > co2[c] + succ[c] + uri[c]	0.3581	-12.8155

22	R01909*	atp[c] + pydxn[c] < = > adp[c] + pdx5p[c]	0.3147	-20.0000

23	R01911*	pi[c] + pydxn[c] < = > h2o[c] + pdx5p[c]	0.2312	-1.0484

24	R02107^$ ^	h2o[c] + o2[c] + xan[c] < = > h2o2[c] + urate[c]	0.2079	-0.1820

25	R05717*	amp[c] + gthox[c] + so3[c] < = > 2 gthrd[c] + aps[c]	0.3186	-20.0000

26	R05794^# ^	chol[c] + cdpdag[c] < = > pc[c] + cmp[c]	0.2051	-0.0002

27	R07171^$ ^	o2[c] + h[c] + nadh[c] < = > h2o2[c] + nad[c]	0.2079	-0.1820

28	R07172^$ ^	o2[c] + h[c] + nadph[c] < = > h2o2[c] + nadp[c]	0.2125	-0.2089

^$ ^Direct formation of oxygen; ^# ^Indirect formation of oxygen through H_2_O_2_; ^# ^Reactions selected for further analysis

Model simulations were also carried out with xylose as the carbon source. The gene candidate identified for anaerobic growth on glucose resulted in a very low biomass flux. This flux was observed when xylose reductase activity was solely dependent on NADH and there was no flux when xylose reductase enzyme used NAPDH or a ratio of NADH and NADPH. *S. stipitis *strain with URA1 gene was not able to grow on xylose under anaerobic conditions [15]. In addition to biosynthetic requirements, further analysis has to be performed on energy, redox balance and regulatory requirements to understand the limitations for anaerobic growth on xylose.

### Xylose utilization by *scheffersomyces stipitis*

**Figure 5:**
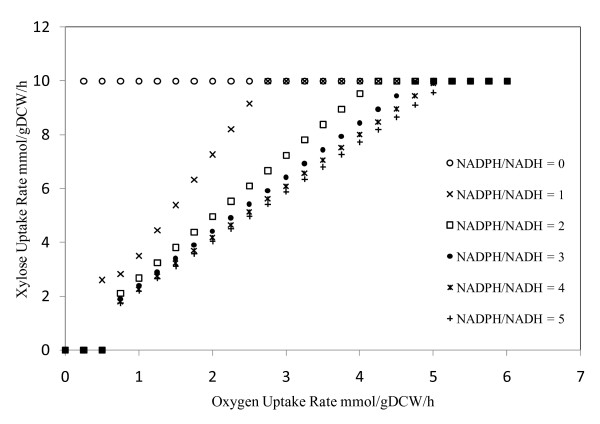
**In silico analysis of xylose uptake**. Dependence of xylose uptake rate on oxygen uptake rate for various NADPH/NADH ratios for xylose reductase.

Xylose is generally utilized by a two-step oxidoreductase reaction catalyzed by xylose reductase and xylitol dehydrogenase. The difference in cofactor specificity between these two reactions often hinders the utilization of xylose and results in the production of xylitol [41,42]. The *S. stipitis *xylose reductase accepts both NADPH and NADH as cofactors with higher preference for NADPH [43] and the xylitol dehydrogenase utilizes NAD as a cofactor. However, xylitol accumulation was found to be negligible in *S. stipitis *[44]. This aspect was analyzed using the model developed for *S. stipitis*. Model simulations were carried out for various ratios of NADPH and NADH dependent xylose reductase and for various uptake rates of xylose and oxygen. No xylitol accumulation was observed in these simulations. The capability of *S. stipitis *to efficiently interconvert NADPH and NADH might be a reason for lower xylitol accumulation. However when the xylose reductase activity was more dependent on NADPH and under lower oxygen uptake rates, it was observed that the xylose uptake rates were limited by oxygen uptake rate (Figure 5). The dependence of xylose uptake rate on oxygen transfer rates has been observed in *S. stipitis *[44] and the substrate consumption rates were improved by having higher initial cell concentration [45].

Reaction insertion analysis used for the identification of metabolic requirements for anaerobic growth was used to identify reactions that enable enhanced uptake of xylose under this condition. Several reactions were able to enhance xylose uptake rate and biomass flux [Data not shown]. A majority of these reactions were able to enhance the uptake rate of xylose by the effective production of the cofactors NADPH and NAD. This was evident when the reversible transdehydrogenase reaction which inter converts NADPH and NAD to NADH and NADP was introduced. This indicates that the metabolic network of *S. stipitis *lacks a sufficient NADPH forming transdehydrogenase reaction.

**Figure 6:**
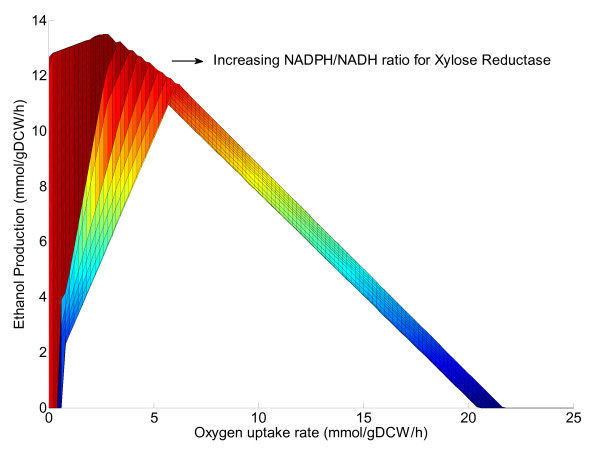
**In silico analysis of ethanol production**. Ethanol production as function of oxygen uptake rate for various NADPH/NADH ratios for xylose reductase. The NADPH/NADH ratio was varied from zero to a very high value (1000000).

The production of ethanol from xylose by *S. stipitis *was analyzed using the model. The existence of an optimal oxygen uptake rate for maximum ethanol yield was observed in the model simulations as reported in literature [14]. A plot of ethanol production rates at various oxygen uptake rates for various ratios of NADPH and NADH dependent xylose reductase activity is shown in Figure 6. It is evident that as the dependency of xylose reductase on NADPH increases, the dependence of optimal ethanol production on oxygen uptake rate also increases.

### Flux variability analysis of the genome scale metabolic model

Flux variability analysis (FVA) was carried out for growth on glucose and xylose using the COBRA toolbox. Reactions known to result in loops within the major metabolic pathways were manually removed from the model before performing the flux variability analysis. The normal FVA calculates the minimum and maximum fluxes across various reactions in major metabolic pathways when maximizing the objective function (Biomass Flux). A variant of FVA called the sub-optimal FVA has been found to be more informative [46], wherein instead of fixing the objective value to an optimal value from the initial FBA, objective lower limit was chosen at 95% of the initial objective value. The normalized flux ranges (normalized with respect to substrate uptake rate) for the major reactions obtained using the suboptimal FVA is shown in supplementary Figures S1-S4 (Additional File 5). Experimental values for the metabolic flux distribution in *S. stipitis *are scarce. The only report on the metabolic flux profiling of *S. stipitis *compared the central carbon metabolism of this yeast with that of *S. cerevisiae *[47]. This data cannot be directly compared with the model simulations as the substrate uptake rates and oxygen transfer rates were not reported.

Analysis of the suboptimal flux variability values for major metabolic pathways in *S. stipitis *metabolic network grown in glucose minimal media revealed a few key reactions which could carry zero flux. One such reaction is phosphoglucose isomerase reaction catalyzed by the pgi1 gene (PICST_84923). Phosphoglucose isomerase pgi1-deletion mutants of *S*. *cerevisiae *cannot grow on glucose as the sole carbon source. The inability of *S. cerevisiae *to efficiently recycle the NADPH generated by the oxidative pentose phosphate pathway has been cited as the major reason for this growth defect [48,49]. However, in *S. stipitis *in silico growth rates were not significantly reduced. In silico metabolic flux analysis was performed for the pgi1-mutant *S. stipitis *to obtain insights on various pathways employed by *S. stipitis *to recycle NADPH generated from oxidative pentose phosphate pathway. Several pathways that could recycle cytosolic NADPH were identified in the *S. stipitis *network. The most promising pathways are listed below

1. NAD-dependent glutamate dehydrogenase and NADP-dependent glutamate dehydrogenase which causes a substrate shuffling between 2-oxoglutarate and glutamate which restores NADP from NADPH through the coupled conversion of NAD to NADH.

2. NADPH dehydrogenase which couples the oxidation of cytoplasmic NADPH to mitochondrial respiratory chain.

3. NAD-dependent alcohol dehydrogenase and NADP-dependent alcohol dehydrogenase which causes a substrate shuffling between ethanol and acetaldehyde which restores NADP from NADPH through the coupled conversion of NAD to NADH.

Comparison of the literature on the phosphoglucose isomerase mutant (pgi1-mutant) *S*. *cerevisiae, Escherichia coli *and *Kluyveromyces lactis *has indicated that the above mentioned pathways are either present in these organisms or when introduced has enhanced the growth of these organisms. *K. lactis *is reported to possess the mitochondrial NADPH dehydrogenase and a transdehydrogenase cycle involving the alcohol dehydrogenase [50,51]. In *E. coli *and *S. cerevisiae *the introduction of a soluble transdehydrogenase gene was found to enhance the growth of pgi1-mutants on glucose [47,52]. Further, in *S. cerevisiae*, over expression of the NAD-dependent glutamate dehydrogenase restored growth in these mutants [49]. From the Biolog phenotype data presented earlier in this paper it can be observed that *S. stipitis *can grow on glutamate as the sole carbon source. The first step in glutamate utilization is NAD-dependent glutamate dehydrogenase and thus this pathway might be responsible for the growth of pgi1-mutant *S. stipitis*. However, all these pathways and the pgi1-mutant strains have to be evaluated experimentally to confirm their roles. Nevertheless, the analysis highlights the usefulness of the metabolic model developed in the designing microbial strains with desired properties.

**Figure 7:**
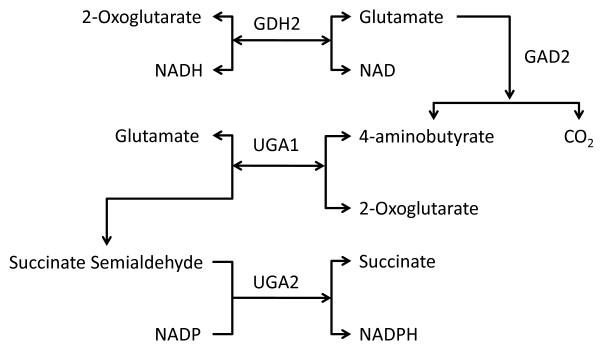
**Cofactor balancing pathway**. Enzymatic reactions which were reported to convert NADH to NADPH in *S. stipitis*. GDH2--NAD-dependent Glutamate dehydrogenase, GAD2--Glutamate decarboxylase, UGA1--4-aminobutyrate aminotransferase (UGA1.1 or UGA1.2) and UGA2--Succinate semialdehyde dehydrogenase (UGA2 or UGA2.2).

Even though *S. stipitis *metabolic network possesses numerous ways for NADPH consumption, the generation of NADPH and NAD to efficiently utilize xylose under lower oxygen uptake rates was limited. One particular pathway was observed to be induced under oxygen limited conditions when the cells are grown on xylose and this pathway was considered to be effective in tackling the cofactor imbalance caused by the first two steps in xylose utilization [25,53]. This pathway involved the four enzymes; NAD-dependent glutamate dehydrogenase (GDH2) which converts 2-oxoglutarate to L-glutamate consuming NADH, glutamate decarboxylase (GAD2) which decarboxylates L-glutamate to 4-aminobutyrate, 4-aminobutyrate aminotransferase (UGA1.1 or UGA1.2) which transaminates 4-aminobutyrate to Succinate semialdehyde and Succinate semialdehyde dehydrogenase (UGA2 or UGA2.2) which oxidizes Succinate semialdehyde to Succinate using NADP (Figure 7). The net result is the conversion of NADH to NADPH. However, the suboptimal flux variability analysis shows that flux through this pathway is limited (maximum of about 35% of substrate uptake rate) and the maximum flux achievable is very low under oxygen limited conditions (about 1-2% of substrate uptake rate) when biomass is maximized. This flux may not be sufficient to generate enough NADPH to increase the substrate uptake rates.

### Mechanism of mitochondrial respiration and oxidative phosphorylation

The role of the model in enhancing the understanding of cellular phenotypes and identifying requirements for metabolism were explained in the previous sections. In addition, the model can also be used to probe mechanisms in metabolism of a microorganism. In this section, the mechanisms involved in mitochondrial respiration and oxidative phosphorylation are analyzed using the model developed.

*S. stipitis *is known to possess a branched respiratory chain which composes of the basic respiratory chain complexes I-IV, an alternative oxidase and alternative NADH dehydrogenases [54]. The effect of inhibition of various complexes in the respiratory chain on the growth of *S. stipitis *has been investigated [55]. Experiments have been carried out to study the role of cytochrome-C oxidase and alternative oxidase on respiration and growth of *S. stipitis *[56-58]. However, a consistent mechanism that explains the mechanism of mitochondrial respiration and oxidative phosphorylation was not immediately evident from these experimental data. To further investigate the mechanism of oxidative phosphorylation in *S. stipitis*, simulations were carried out using the model developed. Simulations were able to predict increase in the ethanol yield observed in a cytochrome-C mutant [57]. However, a simple knockout of individual components of the respiratory chain was not sufficient to explain all the experimental observations reported. The comparison of qualitative model simulations with the experimental data obtained for growth on glucose and xylose is shown in Table 3. The structure of model suggests that all components in the respiratory system are always available and organized into a pathway as needed (when optimized for a particular objective). However this may be not true in the real situation.

**Table 3:** Effect of inhibition of various mitochondrial respiratory complexes on the growth of *Scheffersomyces stipitis *in glucose and xylose. (--) Complete Inhibition; (-) Partial Inhibition; (0) Negligible; (++) Enhanced; NA - Information not available

Complex/Inhibitor	Effect on Growth		Effect on Growth Complex formation AOX and Complex III or IV (Predicted from model analysis)		References
		
	Glucose (*in Vivo*/ In Silico)	Xylose (*in Vivo*/ In Silico)	Glucose (*in Vivo*/ In Silico)	Xylose (*in Vivo*/ In Silico)	
Complex I (Rotenone)	(--/0)	(-/0)	(--/--)	(-/-)	Shi et al., 2002

Complex III (Antimycin A)	(NA/-)	(-/-)	(NA/-)	(-/-)	Lighthelm et al., 1988

AOX (SHAM)	(0/0)	(++/0)	(0/0)	(++/++)	Jeppsson et al., 1995

Complex IV (Cyanide)	(-/-)	(-/-)	(-/-)	(-/-)	Jeppsson et al., 1995

Complex IV and AOX (Sodium Azide)	(NA/--)	--/--)	(NA/--)	--/--)	Lighthelm et al., 1988

Complex IV(Cyanide) and AOX (SHAM)	(--/--)	(--/--)	(--/--)	(--/--)	Jeppsson et al., 1995

**Mutant/Inhibition combination**					

Complex I (Rotenone) + del AOX	(--/0)	(--/0)	(--/--)	(--/--)	Shi et al., 2002

Complex I (Rotenone) + del Complex IV	(--/-)	(--/-)	(--/--)	(--/--)	Shi et al., 2002

## Discussion

One of the well established ways by which microorganisms attain different phenotypic characteristics is by gene regulation. However, in the case of mitochondrial respiration, channeling the electron flow by the formation of super-complexes has been reported as a common mechanism [59-62]. Analysis of the inhibition data on glucose and xylose suggest that either the alternative oxidase or the alternate NADH dehydrogenase may be repressed when grown on glucose. However, studies on cytochrome-C mutants suggest that alternative oxidase may be expressed constitutively [56,57]. Various hypothetical complexes were analyzed through model simulations and were then combined with regulation of gene expression (alternative NADH dehydrogenase) to result in a mechanism which explains all the experimental observations qualitatively. Based on the analysis, the complex formation between alternate oxidase and either Complex III or Complex IV is critical to explain the experimental observations. The proposed mechanism was also able to predict the observed increase in the growth yield in the presence of SHAM (an alternative oxidase inhibitor) during the growth of *S. stipitis *on xylose [56]. This again highlights the utility of model in enhancing our understanding of metabolism of an organism.

An iterative procedure has been designed and used to develop the genome scale metabolic model of *S. stipitis*. The procedure begins with the reconstruction of genome scale metabolic network which is then converted to a fully functional in silico model by incorporating the experimentally determined macromolecular composition, maintenance coefficients and minimal medium requirements. Even though assumption of macromolecular composition based on other related microorganisms or compiling fragmented data from different sources is a common practice [46,63-65], it may lead to improper predictions of essential genes and metabolic flux distribution. Determination of macromolecular composition is critical as it defines the minimal number of metabolites that has to be produced for growth and the relative contribution of these metabolites to growth. Further, it helps in refinement of the genome annotation as some pathways producing these metabolites might not be annotated properly. In the case of *S. stipitis*, higher content of chitin is observed as compared to other yeasts like *S. cerevisiae *(chitin is not a part of the biomass equation used for *S. cerevisiae *genome scale model [33]). Similarly, phosphatidyl-inositol was found in *S. stipitis *biomass but a pathway enzyme which converts myo-inositol to inositol was not annotated in *S. stipitis *genome. A homolog was identified for this enzyme (PICST_63214) and was incorporated into the model. The in silico model was validated for consistency against newly generated high-throughput substrate phenotyping data. The model was subjected to iterative refinements based on identified inconsistencies, leading to additional reactions incorporated into the network and other modifications to the model content. The net result is a biochemically and genetically detailed in silico model that consists of 1371 reactions that are catalyzed by 814 genes. To our knowledge this is the first genome scale metabolic model developed for *S. stipitis *and it qualitatively predicts the phenotypic behaviors for substrate utilization with 74% agreement (252 out of 339 cases).

The model developed has also generated several experimentally verifiable hypotheses that could provide insight into the metabolism of *S. stipitis*. Generation of new annotation for metabolic genes based on network-based gap analysis and high-throughput phenotyping is one such example. Another example is the insight obtained on the mechanism involved in mitochondrial respiratory chain of *S. stipitis*. The results of the inhibition experiments carried out for the various complexes in the mitochondrial respiration chain where explained based on a hypothetical complex formation using in silico metabolic flux analysis. The hypothetical complex formation has to be verified by designing appropriate experiments. Further, the formation of mitochondrial super complexes has already been reported in several yeasts like *S. cerevisiae *and *Yarrowia lipolytica *[61,62].

The predictive capability of the genome scale metabolic network was demonstrated by identifying the biosynthetic requirements for anaerobic growth of *S. stipitis*. The gene insertion analysis performed on the metabolic model was able to identify the particular conversion essential for anaerobic growth on glucose and this was validated by comparing with literature. However, the identified reaction did not enable in silico growth on xylose and this was also observed in experiments [15]. As mentioned in the results sections anaerobic growth is a complex phenotype with biosynthesis complemented by energy, redox balance and regulatory requirements. However, the ability to grow on glucose under anaerobic condition suggests that anaerobic growth on xylose can also be achieved if the other requirements are analyzed systematically. In silico analysis revealed that unconstrained supply of ATP could support anaerobic growth on xylose. Many pathways are known to generate ATP. For example, the bacterial acetate production pathway is commonly employed to improve ATP production [66]. Since acetate has been reported to inhibit xylose fermentation in *S. stipitis *[67], other pathways for ATP generation needs to be investigated. Further redox balance requirements and regulatory requirements should also be considered when developing strategies to promote anaerobic growth.

Xylose uptake analysis carried out using the model indicated that *S. stipitis *metabolic network may not possess sufficient NADPH and/or NAD generation capability under lower oxygen uptake rates. Even though *S. stipitis *is considered to be an efficient xylose utilizing yeast, the metabolic model developed emphasizes the need for further characterization of this yeast to understand and improve its fermentation performance. High throughput techniques routinely used in the characterization of industrial yeasts like *S. cerevisiae *should also be developed for *S. stipitis*. Furthermore, analysis of pgi1-mutant shows that *S. stipitis *metabolic network possess many pathways to consume NADPH and recycle NADP. When grown on glucose the xylose utilization pathway itself can be considered as one such pathway. Thus by performing a detailed analysis on the various NADP recycling pathways and carefully designing suitable mutants the *S. stipitis *metabolic network can be optimized for simultaneous consumption of glucose and xylose. This trait has been a subject of active investigation and is of industrial significance [68].

## Conclusion

In this study, we have reconstructed a genome scale metabolic model for *S. stipitis *by combining information from genome sequence annotation, pathway databases, literature and experimental data. The model was refined using high-throughput phenotyping data. The model predictions were in good agreement with experimental observations, thus allowing us to systematically investigate the physiological characteristics and metabolic capability of this yeast. In silico model analysis shows that *S. stipitis *possesses several pathways to recycle nucleotide cofactors and thus efficient xylose utilization. However, the flux through of these pathways needs experimental investigation. Analysis of mitochondrial respiration and identification of mitochondrial super-complexes demonstrate the novel applications of the model developed. Incorporation of thermodynamic constraints, enzyme kinetics information and high-throughput omics data can further improve the predictability of these models

## Materials and methods

### Metabolic network reconstruction

Figure 1 outlines the overall procedure for reconstruction and validation of the genome scale metabolic model for *S. stipitis*. The entire procedure is based on the recently published protocol for generation of genome scale metabolic models [32]. The reconstruction process was initiated based on annotated genome of *Scheffersomyces stipitis *[25] and ORF (open reading frame) information available on the National Centre for Biological Information http://www.ncbi.nlm.nih.gov/. The biochemical reactions corresponding to the ORFs were mainly compiled from KEGG Database [40,69,70] and MetaCyc Database [71], resulting in a draft reconstruction comprising of enzymes and metabolic reactions.

The next step is the refinement of the draft. For each reaction, gene-protein-reaction association, localization, cofactor specificity, and directionality were identified and assigned. The gene-protein-reaction associations include definitions for isoenzymes and enzyme complexes. The metabolic reactions in the model were organized into three compartments (Cytoplasm, Mitochondria and Extracellular) based on the localization of associated enzymes, which was obtained using the protein localization predictors [72,73] (Lu et al., 2004; Claros and Vincens, 1996). The cofactor and substrate specificity and reaction directionality information were compiled from available literature information, completed genome scale reconstructions [33,63,74-76] and BRENDA database [77,78]. The reactions were then organized into pathways/subsystems. For each metabolite, the charge, formula and identification information were compiled from KEGG database and BIGG database. In the next step, some spontaneous and non-gene associated reactions whose existence was supported by physiological or experimental data from the literature and databases were included. In addition to these reactions, exchange reactions and intracellular and extracellular transport reactions were added. The genes associated with the transport reactions were identified using the transport protein predictor [79]. The final step is the incorporation of biomass equation and, growth and non-growth associated maintenance coefficient.

The final reconstruction was then loaded into Matlab and the in silico model obtained was evaluated for its capability to produce biomass precursors and known by-products from minimal media and gaps in the metabolic network were identified. The gaps were then filled based on pathway databases and published reconstructions. The dead-end reactions in the network were identified and appropriate exchange reactions were added when applicable. The model was then refined and validated iteratively using Biolog phenotyping data. The final model was used for the simulation studies.

### Biomass macromolecular composition estimation

#### Media and cultivation conditions

*Scheffersomyces stipitis *CBS 6054 (CBS6054 = ATCC 58785 = NRRL Y-11545 = IFO 10063) was purchased from American Type Culture Collection. It was routinely cultured in yeast extract, peptone, dextrose (YPD) medium and samples were collected at the exponential growth phase for the estimation of biomass macromolecular composition. Experimental data for biomass composition estimation were collected in duplicates and average values obtained are reported. For growth rate and substrate uptake rate measurements, experiments were carried out in minimal media containing 0.17% yeast nitrogen base without amino acids, 0.5% ammonium sulphate and 2% glucose. Experiments were carried out in 1 liter Erlenmeyer flasks at 30°C and 200 rpm. The OD_600 _was measured periodically for several hours to calculate the growth rates and the supernatant was collected and analyzed using HPLC for calculating the substrate uptake rates. The macromolecular composition of *S*. *stipitis *biomass was estimated by measuring carbohydrate, protein, lipid and nucleic acid content and the components making up these macromolecules.

#### Biomass carbohydrate content

The cell wall polysaccharides (Glucan, Mannan and Chitin) and intracellular carbohydrates (Glycogen and Trehalose) together contribute to the total carbohydrate content. Cell wall isolation and quantification of polysaccharides in the cell wall was carried out as described by Francois, 2007 [80]. Cells were harvested and then lyzed using glass beads in a homogenizer. The cell wall was separated by high speed centrifugation and dried. The supernatant was used for nucleic acid analysis. The dried cell wall was hydrolyzed with 72% sulphuric acid and the sugars liberated were analyzed using HPLC. Glucan and Mannan content was estimated from these liberated sugars. Laminarin and Mannan were used for calibration and galactose was used as the internal standard. For the analysis of chitin content, the cell wall was heated with 6% KOH to liberate chitin. The chitin was treated with chitinase to liberate glucosamine which was detected using Reissig's reagent. Glycogen content was determined as explained by Smolders et al., 1994 [81]. Approximately 20 mg of lyophilised cells was resuspended in 10 ml of 0.6 M HCl and boiled on a heating block at 100°C for 1 h. Glucose liberated from glycogen hydrolysis was quantified using HPLC. For the measurement of trehalose content, cells were washed twice with cold water and resuspended in 3 ml of water for 15 min at 100°C. Sample was incubated overnight with trehalase in 60 ul of acetate buffer. Glucose liberated from trehalose hydrolysis was quantified using HPLC.

#### HPLC analysis of sugars

The sugars glucose and mannose was measured by HPLC using the Biorad Aminex HPX-87H column. 5 mM Sulphuric acid was used as mobile phase and sugars were detected using RID detector operating at 50°C.

#### Biomass protein and amino acid content

Total protein content of *S. stipitis *biomass is determined using nitric acid method [82]. The harvested cells were washed in TE buffer and centrifuged. The cells were then solubilized in 70% nitric acid and incubated at 22°C for 24 h. The absorbance was measured at 358 nm to obtain the protein content. Calibration curves for protein estimation were obtained using BSA. The amino acid content of *S. stipitis *biomass was measured using standard protocols as explained in AOAC Official Method 994.12 and AOAC Official Method 985.28 [83].

#### Biomass nucleic acid content

The DNA and RNA content was determined using Orcinol reagent (0.1% Orcinol, 0.1% FeCl_3_.6H_2_O in concentrated HCl) [84]. Freeze-dried cell lysate was resuspended and diluted in autoclaved MilliQ water for DNA and RNA analysis. Equal volumes of diluted sample and freshly prepared Orcinol reagent were mixed and incubated at 100°C for either 2 min (for DNA estimation) or 15 min (Total nucleic acid estimation). The mixtures were immediately cooled on ice. The mixture incubated for 2 min was further incubated at 37°C for 2 h after cooling on ice. The absorbance was measured at 600 nm to obtain the DNA and total nucleic acid content. The values obtained from DNA measurements were subtracted from the total nucleic acid values to obtain the RNA content of the cell lysate. Calibration curves were obtained using standard DNA and RNA.

#### Biomass lipid content

The total lipid content was determined as explained by Matyash et al., 2008 [85]. Harvested cells were washed twice with 10 ml of 0.1% ammonium acetate solution and resuspended in 6 ml of ammonium acetate solution. Optical density was measured and 5 ml of cell suspension was added to 7.5 ml of methanol and vortexed vigorously. 25 ml of methyl-tert-butyl ether was added and the mixture was shaken at 250 rpm for 1 h. Cell debris was removed by filtration and 6.25 ml of water was added to the mixture. The organic phase was extracted twice with the solvent mixture and dried in a pre-weighed round bottom flask. Lipid content was then calculated based on the weight of the lipids extracted. The individual lipid classes were measured using TLC. Polar lipids were separated on a silica gel TLC plate with chloroform/methanol/water (65:25:4) as an eluent as described by Skipski et al., 1962 [86]. Neutral lipids were separated on a silica gel TLC plate with hexane/Diethyl ether/formic acid (45:5:1) as an eluent as described by Low et al., 2009 [87]. The separated lipids were quantified by densitometry using standard lipids. The fatty acid composition of *S. stipitis *was obtained using standard protocols as explained in AOAC Official Method 996.06 [83].

The composition of the various biomass macromolecules estimated were converted to a biomass synthesis equation to be incorporated into to model developed. The growth and substrates uptake data estimated in the present study, along with reported data from continuous cultivation experiments [88] were used to calculated the growth and non-growth associated maintenance coefficients (Refer to Additional File 1 for details).

### In silico computations

The metabolic network was loaded into Matlab using functions available in the COBRA toolbox [89,90]. The metabolic capabilities of *S. stipitis *network were calculated by using flux balance analysis and linear optimization. For growth simulations, biomass synthesis was selected as the objective to be maximized and the optimization was solved using the COBRA Toolbox. The external metabolites were allowed to freely cross the system boundary by having unconstrained exchange reaction. Units of all flux values are in mmol/gDCW/h. For the simulation of aerobic growth on minimal media, the following external metabolites were allowed to freely enter and leave the network: NH_4_, O_2_, H+, SO_4_, PO_4_, CO_2 _and H_2_O (exchange fluxs -1000-1000 mmol/gDCW/h). All other external metabolites, except the substrates tested were only allowed to leave the system (exchange fluxes 0-1000 mmol/gDCW/h). Growth on different substrates was simulated by allowing the corresponding external metabolite to enter the system with the definite exchange rate (10 mmol/gDCW/h). For the simulation of anaerobic growth, oxygen uptake was reduced to zero and unconstrained uptake of sterols and unsaturated fatty acids was allowed.

### Phenotype microarray analysis of *scheffersomyces stipitis*

Biolog's Phenotype MicroArray™ technology [34] was used for the phenotypic analysis of *Scheffersomyces stipitis*. It permits assays of 190 carbon (PM1- and PM2A- microplates), 95 nitrogen (PM3BMicroplate), 59 phosphorus and 35 sulphur-source (PM4A-Microplate) utilizations at once. A defined medium containing 100 mM glucose, 5.0 mM NH4Cl, 2.0 mM NaH2PO4, 0.25 mM Na2SO4, 100 mM NaCl, 30 mM triethanolamine HCl (pH 7.1), 0.05 mM MgCl2, 1.0 mM KCl, 1.0 mM FeCl3, and 0.01% tetrazolium violet was used for the PM tests. The PM plates contained various carbon-, nitrogen-, phosphorus-, or sulphur-sources which are omitted from the defined medium. The microplates were incubated at 30°C and the dye reduction data were collected in 15-min intervals for 48 h. In addition to time profile dye reduction data, the absorbance was measured at 590 nm and 750 nm after 24 h and 48 h incubations. The colorimetric assay was considered positive when the absorbance corresponding to reduced dye was 1.2 times higher than the negative control. Similar threshold was applied for the absorbance at 750 nm. The threshold was set at 1.3 for sulphur source data as the negative control was observed to have a higher background. The confidence with which growth can be predicted based on these measurements was determined based on the number of positive reactions out of 4 absorbance measurements. High confidence growth (+) if all measurements were above threshold and high confidence no-growth (-) if below. The remaining cases were classified as low-confidence data.

## Competing interests

The authors declare that they have no competing interests.

## Authors' contributions

BB and SJ designed the model development framework. BB executed the model development work. LT and BB designed the experiments and collected the data. BB and SJ involved in the analysis of results. RS supervised the study and assisted in implementation. BB drafted the manuscript. All the authors read and approved the final manuscript.

## Supplementary Material

Additional file 1 **Estimation of growth and non-growth associated maintenance requirements**. Click here for file

Additional file 2 **Details of the reactions and metabolites in the genome scale metabolic model of *Scheffersomyces stipitis***. Click here for file

Additional file 3 **List of essential reactions in the genome scale model**. Click here for file

Additional file 4 **Comparison of model predictions and Biolog substrate utilization data**. The details of substrates tested, abbreviations used for these substrates in the model, additional experimental evidence and model refinements are described in this file. Click here for file

Additional file 5 **Sub-Optimal Flux variability analysis of *Scheffersomyces stipitis *metabolic model (Figures S1-S4)**. Click here for file
