# Supplementary material for: Reconstruction and analysis of a genome-scale metabolic model for Scheffersomyces stipitis
Source: Microb Cell Fact. 2012 Feb 23;11:27. doi: 10.1186/1475-2859-11-27 (PMC3310799; doi:10.1186/1475-2859-11-27)
Supplement: Additional file 1 — Estimation of growth and non-growth associated maintenance requirements. [file 1475-2859-11-27-S1.PDF]

## Calculation of growth and non-growth associated maintenance requirements for *Sc. stipitis*

The growth and non-growth associated maintenance coefficients (GAM and NGAM) for *Sc. stipitis* were estimated by plotting the glucose uptake rate against the growth rate. Data obtained from our experiments and that reported in literature (van Urk et al, 1990) was used. Model simulations were carried out for various values of GAM and the results were plotted with experimental data as shown in figure below. GAM of 44.4 was found to match with experimental data. The NGAM was calculated from the y-intercept. By maximizing ATP turnover under the glucose uptake constraint of 1 mmol/gDCW-hr, the ATP yield is evaluated as $Y_{\mathrm{ATP,\ max}}$ = 28.6 mol ATP/ mol glucose. Using this value and the y-intercept (0.095 mmol glucose/gDCW-hr), we can calculate the NGAM requirement to be about 2.7 mmol ATP/gDCW/hr

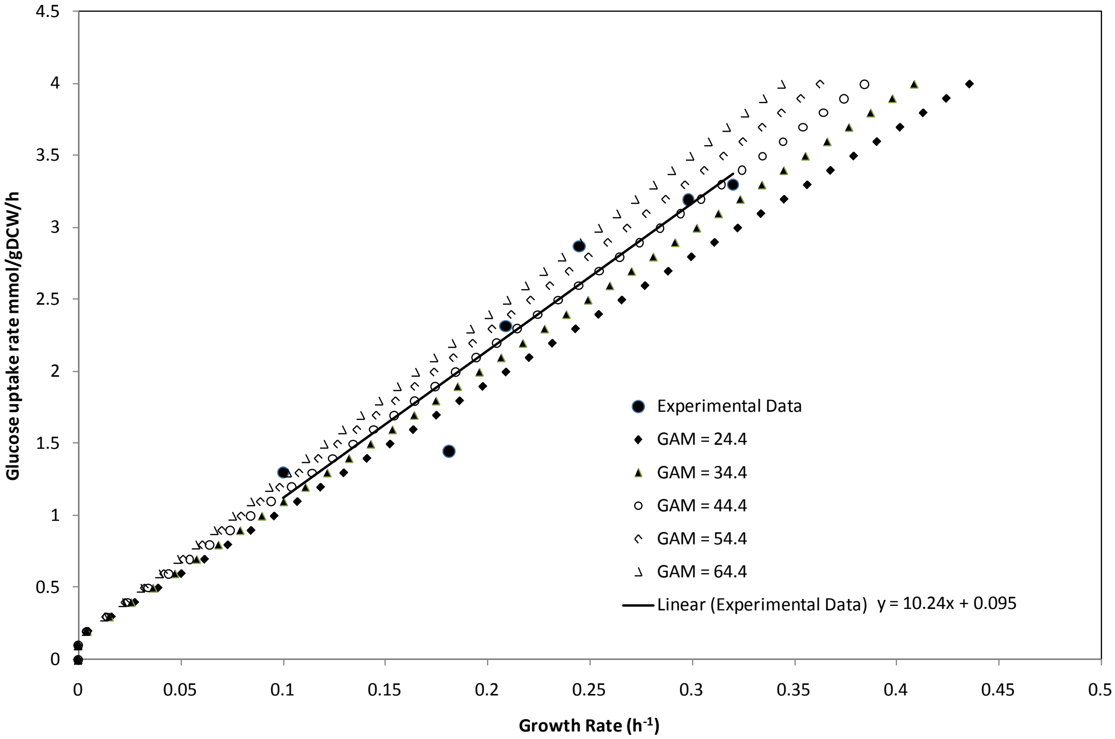


## References

van Urk H, Voll WSL, Scheffers WA, van Dijken JP (1990) Transient-state analysis of metabolic fluxes in crabtree-positive and crabtree-negative yeasts. *Appl Microbiol Biotechnol* **56(1)**: 281-287.
