# Supplementary material for: Reconstruction and analysis of a genome-scale metabolic model for Scheffersomyces stipitis
Source: Microb Cell Fact. 2012 Feb 23;11:27. doi: 10.1186/1475-2859-11-27 (PMC3310799; doi:10.1186/1475-2859-11-27)
Supplement: Additional file 3 — List of essential reactions in the genome scale model. [file 1475-2859-11-27-S3.PDF]

List of Essential Reactions

S.No	Reaction Name	Reaction Formula
1	13GS	udpg[c] -> 13BDglcn[c] + h[c] + udp[c]
2	2OXOADPtm	2oxoadp[m] -> 2oxoadp[c]
3	3C3HMPtm	3c3hmp[c] <=> 3c3hmp[m]
4	AATA	2oxoadp[c] + glu-L[c] <=> akg[c] + L2aadp[c]
5	ACGAM6PS	accoa[c] + gam6p[c] <=> h[c] + coa[c] + acgam6p[c]
6	ACGAMPM	acgam6p[c] <=> acgam1p[c]
7	ACGAMPP	h[c] + acgam1p[c] + utp[c] <=> ppi[c] + uacgam[c]
8	ACGKm	atp[m] + acglu[m] -> adp[m] + acg5p[m]
9	ACLSm	h[m] + 2 pyr[m] -> co2[m] + alac-S[m]
10	ACONHm	cit[m] <=> icit[m]
11	ACOTAm	glu-L[m] + acg5sa[m] -> acorn[m] + akg[m]
12	ADSK	atp[c] + aps[c] -> h[c] + adp[c] + paps[c]
13	ADSL1	dcamp[c] <=> amp[c] + fum[c]
14	ADSL2	25aics[c] <=> fum[c] + aicar[c]
15	ADSS	asp-L[c] + gtp[c] + imp[c] -> 2 h[c] + pi[c] + dcamp[c] + gdp[c]
16	AGAT	0.028 hexacoa[c] + 0.023 octacoa[c] + 0.019 dcacoa[c] + 0.115 ddcacoa[c] + 0.058 tdcoa[c] + 0.18 pmtcoa[c] + 0.078 hdceacoa[c] + 0.035 stcoa[c] + 0.315 ocdceacoa[c] + 0.094 ocdcyacoa[c] + 0.012 ocdctacoa[c] + 0.011 eicosapencoa[c] + 0.01 docosacoa[c] + 0.01 1ag3p[c] -> coa[c] + 0.01 pa[c]
17	AGPRm	h[m] + acg5p[m] + nadph[m] -> pi[m] + acg5sa[m] + nadp[m]
18	AHC	h2o[c] + ahcys[c] -> adn[c] + hcys-L[c]
19	AICART	aicar[c] + 10fthf[c] <=> fprica[c] + thf[c]
20	AIRC	co2[c] + air[c] <=> h[c] + 5aizc[c]
21	ANPRT	prpp[c] + anth[c] -> ppi[c] + pran[c]
22	ANS	chor[c] + gln-L[c] -> h[c] + glu-L[c] + pyr[c] + anth[c]
23	ARGSL	argsuc[c] <=> fum[c] + arg-L[c]
24	ARGSS	atp[c] + asp-L[c] + citr-L[c] <=> h[c] + amp[c] + ppi[c] + argsuc[c]
25	ASNS1	h2o[c] + atp[c] + gln-L[c] + asp-L[c] -> h[c] + glu-L[c] + amp[c] + ppi[c] + asn-L[c]
26	ASPCT	asp-L[c] + cbp[c] -> h[c] + pi[c] + cbasp[c]
27	ATPPRT	atp[c] + prpp[c] -> ppi[c] + prbatp[c]
28	C14STR	h[c] + nadph[c] + 44mctr[c] -> nadp[c] + 44mzym[c]
29	C24STR	h[c] + nadph[c] + ergtetrol[c] -> nadp[c] + ergst[c]
30	C3STDH1	nad[c] + 4mzym_int1[c] -> h[c] + co2[c] + nadh[c] + 4mzym_int2[c]
31	C3STDH2	nad[c] + zym_int1[c] -> h[c] + co2[c] + nadh[c] + zym_int2[c]
32	C3STKR1	h[c] + nadph[c] + 4mzym_int2[c] -> nadp[c] + 4mzym[c]
33	C3STKR2	h[c] + nadph[c] + zym_int2[c] -> nadp[c] + zymst[c]
34	C4STMO1	3 h[c] + 3 o2[c] + 3 nadph[c] + 44mzym[c] -> 4 h2o[c] + 3 nadp[c] + 4mzym_int1[c]

35	C4STMO2	3 h[c] + 3 o2[c] + 3 nadph[c] + 4mzym[c] -> 4 h2o[c] + 3 nadp[c] + zym_int1[c]
36	C5STDS	h[c] + o2[c] + nadph[c] + epist[c] -> 2 h2o[c] + nadp[c] + ergtrol[c]
37	C8STI	fecost[c] -> epist[c]
38	CBPS	h2o[c] + 2 atp[c] + hco3[c] + gln-L[c] -> 2 h[c] + glu-L[c] + 2 adp[c] + pi[c] + cbp[c]
39	CHORM	chor[c] -> pphn[c]
40	CHORS	3psme[c] -> pi[c] + chor[c]
41	CHTNS	uacgam[c] -> h[c] + udp[c] + chitin[c]
42	CITSm	h2o[m] + accoa[m] + oaa[m] -> h[m] + coa[m] + cit[m]
43	CITtam	cit[c] + mal-L[m] <=> cit[m] + mal-L[c]
44	CITtcm	icit[m] + cit[c] <=> cit[m] + icit[c]
45	CO2tm	co2[c] <=> co2[m]
46	CTPtm	2 h[c] + cmp[m] + ctp[c] -> 2 h[m] + cmp[c] + ctp[m]
47	CYSS	acser[c] + h2s[c] -> h[c] + ac[c] + cys-L[c]
48	CYSTL	h2o[c] + cyst-L[c] -> nh4[c] + pyr[c] + hcys-L[c]
49	DADK	atp[c] + damp[c] <=> adp[c] + dadp[c]
50	DASYN	h[c] + 0.01 pa[c] + ctp[c] <=> ppi[c] + 0.01 cdpdag[c]
51	DASYNm	h[m] + ctp[m] + 0.01 pa[m] <=> ppi[m] + 0.01 cdpdag[m]
52	DDPA	h2o[c] + pep[c] + e4p[c] -> pi[c] + 2dda7p[c]
53	DHAD1m	23dhmb[m] -> h2o[m] + 3mob[m]
54	DHFRi	h[c] + nadph[c] + dhf[c] -> nadp[c] + thf[c]
55	DHORD	o2[c] + dhor-S[c] -> h2o2[c] + orot[c]
56	DHORTS	h2o[c] + dhor-S[c] <=> h[c] + cbasp[c]
57	DHQS	2dda7p[c] -> pi[c] + 3dhq[c]
58	DHQT	3dhq[c] -> h2o[c] + 3dhsk[c]
59	DMATT	dmpp[c] + ipdp[c] -> ppi[c] + grdp[c]
60	DOLPMMer	dolmanp[c] -> h[c] + mannan[c] + dolp[c]
61	DOLPMTcer	dolp[c] + gdpmann[c] -> gdp[c] + dolmanp[c]
62	DPMVD	atp[c] + 5dpmev[c] -> co2[c] + adp[c] + pi[c] + ipdp[c]
63	EX_nh4(e)	nh4[e] <=>
64	EX_o2(e)	o2[e] <=>
65	EX_pi(e)	pi[e] <=>
66	EX_so4(e)	so4[e] <=>
67	G3PD1	h[c] + nadh[c] + dhap[c] -> nad[c] + glyc3p[c]
68	G5SAD	glu5sa[c] <=> h2o[c] + h[c] + 1pyr5c[c]
69	GALU	h[c] + utp[c] + g1p[c] <=> udpg[c] + ppi[c]
70	GARFT	10fthf[c] + gar[c] -> h[c] + thf[c] + fgam[c]
71	GAT1	0.028 hexacoa[c] + 0.023 octacoa[c] + 0.019 dcacoa[c] + 0.115 ddcacoa[c] + 0.058 tdcoa[c] + 0.18 pmtcoa[c] + 0.078 hdceacoa[c] + 0.035 stcoa[c] + 0.315 ocdceacoa[c] + 0.094 ocdcyacoa[c] + 0.012 ocdctacoa[c] + 0.011 eicosapencoa[c] + 0.01 docosacoa[c] + glyc3p[c] -> coa[c] + 0.01 1ag3p[c]
72	GF6PTA	gln-L[c] + f6p[c] -> glu-L[c] + gam6p[c]

73	GK3	atp[c] + dgmp[c] <=> adp[c] + dgdp[c]
74	GLNS	glu-L[c] + atp[c] + nh4[c] -> h[c] + adp[c] + pi[c] + gln-L[c]
75	GLUPRT	h2o[c] + gln-L[c] + prpp[c] -> glu-L[c] + ppi[c] + pram[c]
76	GMPS	h2o[c] + atp[c] + gln-L[c] + xmp[c] -> 2 h[c] + glu-L[c] + amp[c] + ppi[c] + gmp[c]
77	GRTT	ipdp[c] + grdp[c] -> ppi[c] + frdp[c]
78	HCITSm	h2o[m] + accoa[m] + akg[m] -> h[m] + coa[m] + hicit[m]
79	HCO3E	h2o[c] + co2[c] <=> h[c] + hco3[c]
80	HICITDm	nad[m] + hicit[m] <=> h[m] + nadh[m] + oxag[m]
81	HISTD	h2o[c] + 2 nad[c] + histd[c] -> 3 h[c] + 2 nadh[c] + his-L[c]
82	HISTP	h2o[c] + hisp[c] -> pi[c] + histd[c]
83	HMGCOAR	2 nadp[c] + coa[c] + mev-R[c] <=> 2 h[c] + 2 nadph[c] + hmgcoa[c]
84	HSTPT	glu-L[c] + imacp[c] -> akg[c] + hisp[c]
85	ICDH1	nadp[c] + icit[c] -> co2[c] + nadph[c] + akg[c]
86	IG3PS	gln-L[c] + prlp[c] -> h[c] + glu-L[c] + aicar[c] + eig3p[c]
87	IGPDH	eig3p[c] -> h2o[c] + imacp[c]
88	IGPS	h[c] + 2cpr5p[c] -> h2o[c] + co2[c] + 3ig3p[c]
89	IMPC	h2o[c] + imp[c] <=> fprica[c]
90	IMPD	h2o[c] + nad[c] + imp[c] -> h[c] + nadh[c] + xmp[c]
91	IPDDI	ipdp[c] <=> dmpp[c]
92	IPMD	nad[c] + 3c2hmp[c] -> h[c] + 3c4mop[c] + nadh[c]
93	IPPMIa	3c2hmp[c] <=> h2o[c] + 2ippm[c]
94	IPPMIb	h2o[c] + 2ippm[c] <=> 3c3hmp[c]
95	IPPS	h2o[m] + 3mob[m] + accoa[m] -> h[m] + 3c3hmp[m] + coa[m]
96	KARA1m	h[m] + alac-S[m] + nadph[m] -> nadp[m] + 23dhmb[m]
97	LEUTA	akg[c] + leu-L[c] <=> glu-L[c] + 4mop[c]
98	LNS14DM	2 h[c] + 3 o2[c] + 3 nadph[c] + lanost[c] -> 4 h2o[c] + 3 nadp[c] + 44mctr[c] + for[c]
99	LNSTLS	Ssq23epx[c] -> lanost[c]
100	MAN1PGT	h[c] + gtp[c] + man1p[c] -> ppi[c] + gdpmann[c]
101	MAN6PI	man6p[c] <=> f6p[c]
102	METAT	h2o[c] + atp[c] + met-L[c] -> ppi[c] + pi[c] + amet[c]
103	METS	hcys-L[c] + 5mthf[c] -> met-L[c] + thf[c]
104	MFAPS	amet[c] + 0.01 ptdmeeta[c] -> h[c] + ahcys[c] + 0.01 ptd2meeta[c]
105	MI1PP	h2o[c] + mi1p-D[c] -> pi[c] + inost[c]
106	MI1PS	g6p[c] -> mi1p-D[c]
107	MTHFR2	2 h[c] + nadph[c] + mlthf[c] -> nadp[c] + 5mthf[c]
108	NDPK2	udp[c] + atp[c] <=> adp[c] + utp[c]
109	NH4t	nh4[e] <=> nh4[c]
110	O2t	o2[e] <=> o2[c]
111	OCBT	orn[c] + cbp[c] -> h[c] + pi[c] + citr-L[c]
112	OMCDC	h[c] + 3c4mop[c] -> co2[c] + 4mop[c]
113	OMPDC	h[c] + orot5p[c] -> co2[c] + ump[c]

114	ORNTACim	glu-L[m] + acorn[m] -> acglu[m] + orn[m]
115	ORNtm	h[c] + orn[m] <=> h[m] + orn[c]
116	ORPT	ppi[c] + orot5p[c] <=> prpp[c] + orot[c]
117	OXAGm	h[m] + oxag[m] <=> 2oxoadp[m] + co2[m]
118	P5CR	2 h[c] + nadph[c] + 1pyr5c[c] -> nadp[c] + pro-L[c]
119	PAPSR	paps[c] + trdrd[c] -> 2 h[c] + pap[c] + so3[c] + trdox[c]
120	Patm	pa[c] <=> pa[m]
121	PETOHM	amet[c] + 0.01 pe[c] -> h[c] + ahcys[c] + 0.01 ptdmeeta[c]
122	PGMT	g1p[c] <=> g6p[c]
123	PHETA1	akg[c] + phe-L[c] <=> glu-L[c] + phpyr[c]
124	PINOS	0.01 cdpdag[c] + inost[c] -> h[c] + cmp[c] + 0.01 ptd1ino[c]
125	PIt2r	h[e] + pi[e] <=> h[c] + pi[c]
126	PMANM	man1p[c] <=> man6p[c]
127	PMETM	amet[c] + 0.01 ptd2meeta[c] -> h[c] + ahcys[c] + 0.01 pc[c]
128	PMEVK	atp[c] + 5pmev[c] -> adp[c] + 5dpmev[c]
129	PPAm	h2o[m] + ppi[m] -> h[m] + 2 pi[m]
130	PPND	nadp[c] + pphn[c] -> 34hpp[c] + co2[c] + nadph[c]
131	PPNDH	h[c] + pphn[c] -> h2o[c] + co2[c] + phpyr[c]
132	PRAGS	atp[c] + gly[c] + pram[c] <=> h[c] + adp[c] + pi[c] + gar[c]
133	PRAI	pran[c] -> 2cpr5p[c]
134	PRAIS	atp[c] + fpram[c] -> 2 h[c] + adp[c] + pi[c] + air[c]
135	PRAMPC	h2o[c] + prbamp[c] -> prfp[c]
136	PRASCS	atp[c] + asp-L[c] + 5aizc[c] <=> h[c] + adp[c] + pi[c] + 25aics[c]
137	PRATPP	h2o[c] + prbatp[c] -> h[c] + ppi[c] + prbamp[c]
138	PRFGS	h2o[c] + atp[c] + gln-L[c] + fgam[c] -> h[c] + glu-L[c] + adp[c] + pi[c] + fpram[c]
139	PRMICI	prfp[c] -> prlp[c]
140	PRPPS	atp[c] + r5p[c] <=> h[c] + amp[c] + prpp[c]
141	PSCVT	pep[c] + skm5p[c] -> pi[c] + 3psme[c]
142	PSERSm	0.01 cdpdag[m] + ser-L[m] <=> h[m] + cmp[m] + 0.01 ps[m]
143	RPI	r5p[c] <=> ru5p-D[c]
144	SACCD1	h[c] + nadph[c] + glu-L[c] + L2aadp6sa[c] <=> h2o[c] + nadp[c] + saccrp-L[c]
145	SACCD2	h2o[c] + nad[c] + saccrp-L[c] <=> h[c] + akg[c] + nadh[c] + lys-L[c]
146	SADT	h[c] + atp[c] + so4[c] -> ppi[c] + aps[c]
147	SAM24MT	amet[c] + zymst[c] -> h[c] + ahcys[c] + fecost[c]
148	SERAT	accoa[c] + ser-L[c] -> coa[c] + acser[c]
149	SHK3D	h[c] + nadph[c] + 3dhsk[c] -> nadp[c] + skm[c]
150	SHKK	atp[c] + skm[c] -> h[c] + adp[c] + skm5p[c]
151	SHSL1	cys-L[c] + suchms[c] -> h[c] + cyst-L[c] + succ[c]
152	SHSL4	h2o[c] + suchms[c] <=> h[c] + 2obut[c] + nh4[c] + succ[c]
153	SO4t	so4[e] -> so4[c]
154	SQLE	h[c] + o2[c] + nadph[c] + sql[c] -> h2o[c] + nadp[c] + Ssq23epx[c]

155	SQLS	h[c] + nadph[c] + 2 frdp[c] -> nadp[c] + 2 ppi[c] + sql[c]
156	SULRy	3 h2o[c] + 3 nadp[c] + h2s[c] <=> 5 h[c] + 3 nadph[c] + so3[c]
157	TMDS	dump[c] + mlthf[c] -> dtmp[c] + dhf[c]
158	TRDR	h[c] + nadph[c] + trdox[c] -> nadp[c] + trdrd[c]
159	TRE6PP	h2o[c] + tre6p[c] -> pi[c] + tre[c]
160	TRE6PS	udpg[c] + g6p[c] -> h[c] + udp[c] + tre6p[c]
161	TRPS1	ser-L[c] + 3ig3p[c] -> h2o[c] + trp-L[c] + g3p[c]
162	UMPK	atp[c] + ump[c] <=> udp[c] + adp[c]
