# Supplementary material for: Reconstruction and analysis of a genome-scale metabolic model for Scheffersomyces stipitis
Source: Microb Cell Fact. 2012 Feb 23;11:27. doi: 10.1186/1475-2859-11-27 (PMC3310799; doi:10.1186/1475-2859-11-27)
Supplement: Additional file 5 — Sub-Optimal Flux variability analysis of Scheffersomyces stipitis metabolic model (Figures S1-S4). [file 1475-2859-11-27-S5.PDF]

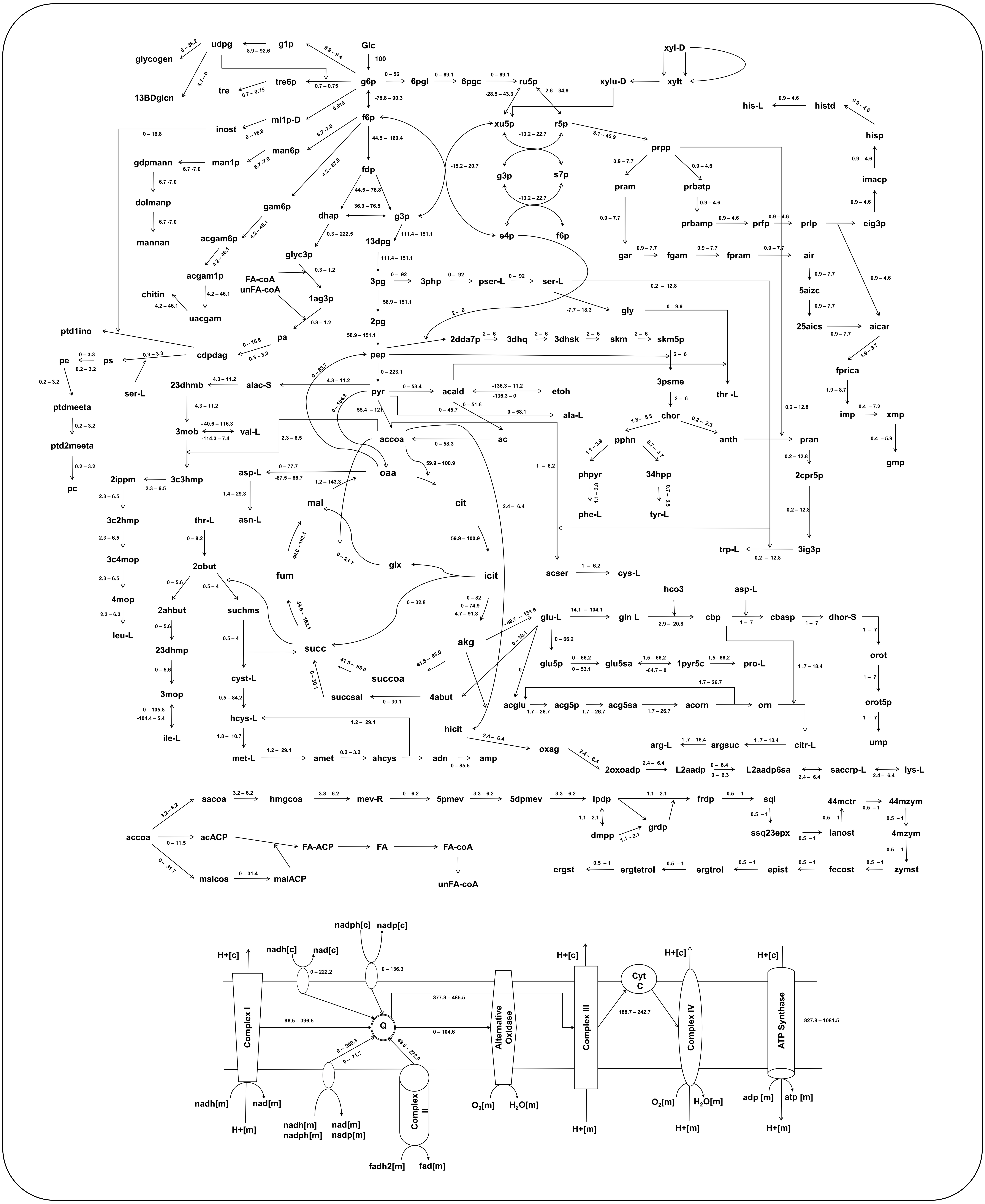

Supplementary Figure S1. Sub-Optimal Flux Variability Analysis for growth on Glucose

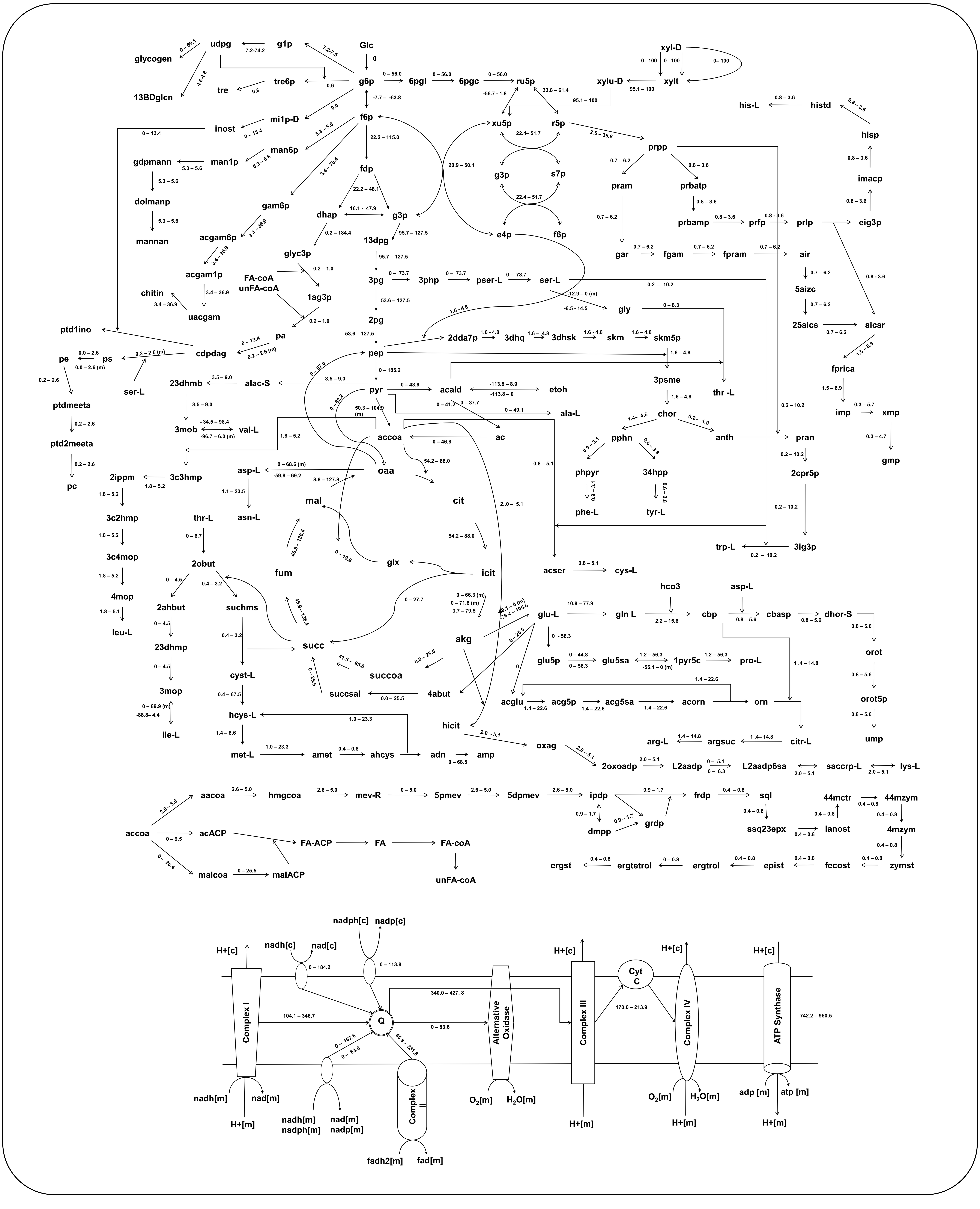

Supplementary Figure S2. Sub-Optimal Flux Variability Analysis for growth on Xylose (NADH preferring)

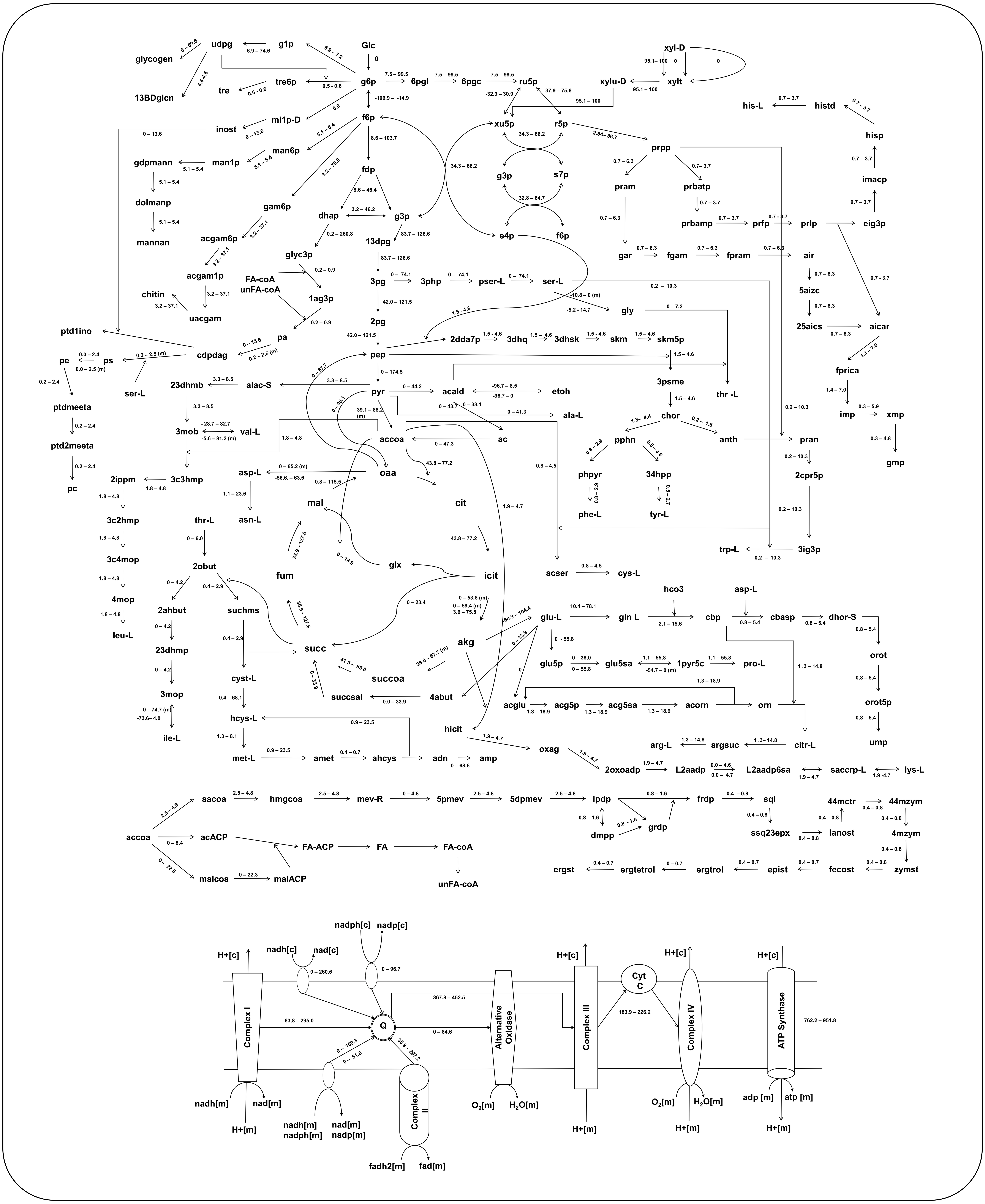

Supplementary Figure S3. Sub-Optimal Flux Variability Analysis for growth on Xylose (NADPH preferring)

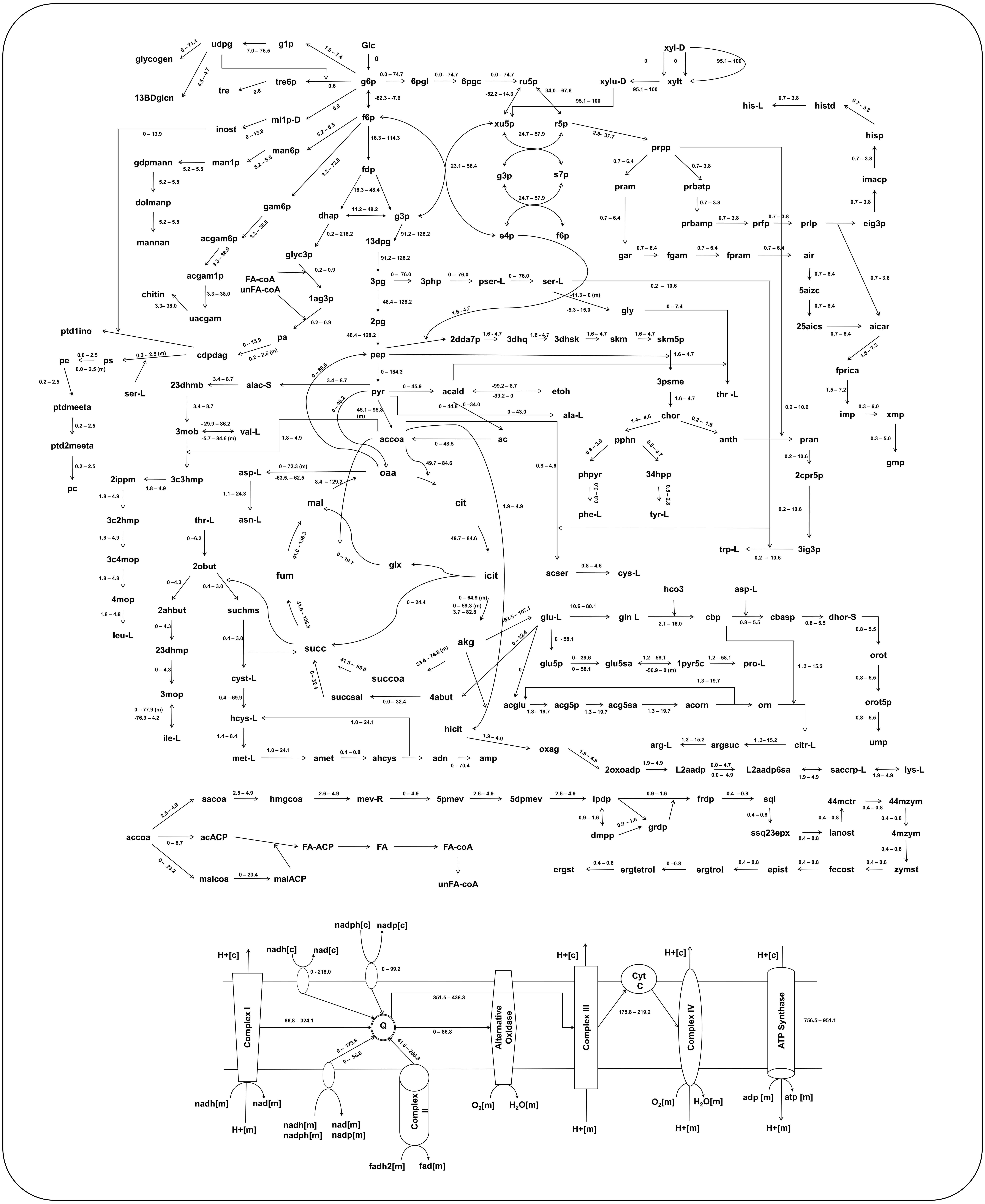

Supplementary Figure S4. Sub-Optimal Flux Variability Analysis for growth on Xylose (1:1 NADH/NADPH)
